# The capacity of primary healthcare facilities in Bangladesh to prevent and control non-communicable diseases

**DOI:** 10.1186/s12875-023-02016-6

**Published:** 2023-03-02

**Authors:** Ashraful Kabir, Md Nazmul Karim, Baki Billah

**Affiliations:** Department of Epidemiology and Preventive Medicine, School of Public Health and Preventive Medicine, Monash University, Melbourne, Australia

**Keywords:** Bangladesh, Health system, Non-communicable diseases, Primary healthcare facilities, Service Availability and Readiness Assessment, WHO SARA

## Abstract

**Background:**

The rapid rise of non-communicable diseases (NCDs) has become a significant public health concern in Bangladesh. This study assesses the readiness of primary healthcare facilities to manage the following NCDs: diabetes mellitus (DM), cervical cancer, chronic respiratory diseases (CRIs), and cardiovascular diseases (CVDs).

**Methods:**

A cross-sectional survey was conducted between May 2021 and October 2021 among 126 public and private primary healthcare facilities (nine Upazila health complexes (UHCs), 36 union-level facilities (ULFs), 53 community clinics (CCs), and 28 private hospitals/clinics). The NCD-specific service readiness was assessed using the World Health Organization’s (WHO) Service Availability and Readiness Assessment (SARA) reference manual. The facilities’ readiness was assessed using the following four domains: guidelines and staff, basic equipment, diagnostic facility, and essential medicine. The mean readiness index (RI) score for each domain was calculated. Facilities with RI scores of above 70% were considered ‘ready’ to manage NCDs.

**Results:**

The general services availability ranged between 47% for CCs and 83% for UHCs and the guidelines and staff accessibility were the highest for DM in the UHCs (72%); however, cervical cancer services were unavailable in the ULFs and CCs. The availability of basic equipment was the highest for cervical cancer (100%) in the UHCs and the lowest for DM (24%) in the ULFs. The essential medicine for CRI was 100% in both UHCs and ULFs compared to 25% in private facilities. The diagnostic capacity for CVD and essential medicine for cervical cancer was unavailable at all levels of public and private healthcare facilities. The overall mean RI for each of the four NCDs was below the cut-off value of 70%, with the highest (65%) for CRI in UHCs but unavailable for cervical cancer in CCs.

**Conclusion:**

All levels of primary healthcare facilities are currently not ready to manage NCDs. The notable deficits were the shortage of trained staff and guidelines, diagnostic facilities, and essential medicine. This study recommends increasing service availability to address the rising burden of NCDs at primary healthcare levels in Bangladesh.

## Background

Non-communicable diseases (NCDs) have become the leading cause of morbidity and mortality worldwide [1, 2]. NCDs are attributable to 71% of total deaths (41 million) globally, and 80% of these deaths occur in low- and middle-income countries [1]. If the current trend continues, the annual cumulative deaths from NCDs will reach 52 million by 2030 [3]. In acknowledgment of the pace of the increasing burden of NCDs, the World Health Organization (WHO) formulated the Global Action Plan (2013–2020) for the prevention and control of NCDs [4]. One of the key features of this global roadmap is to strengthen and refocus the health system to manage the four NCDs, namely diabetes mellitus (DM), cancer, chronic respiratory diseases (CRIs), and cardiovascular diseases (CVDs) by accelerating the provision of equitable and affordable access to service and care [5, 6]. Many countries across the globe have endorsed this strategy and subsequently designed a set of actions in line with their national goals and/or policy priorities to facilitate the provision of NCDs in primary healthcare settings [7–10]. Several studies were conducted to assess the readiness of healthcare facilities in terms of the availability of various components (e.g., infrastructure, workforce, logistics and supplies, guidelines, and protocols) at different healthcare levels (e.g., primary, secondary, tertiary) [11–15].

Despite the substantial progress in overall health outcomes over the past decades, NCDs have become a severe public health concern in Bangladesh [16–18]. According to the latest data, the estimated number of deaths from NCDs was 67% of the total deaths in 2016, which shared approximately 64% of the disease burden in the country [19]. Currently, NCDs present substantial challenges to the country’s resource-constrained healthcare system. The burden of NCDs is projected to be intensified in the future due to the increasing elderly population, rapid urbanization, and lifestyle factors such as tobacco use, alcohol consumption, physical inactivity, and unhealthy diets [20, 21]. Additionally, insufficient healthcare facilities and infrastructure and the shortage of trained healthcare staff will likely exacerbate the burden of NCDs, which will result in remarkable pressure on the healthcare system [22, 23].

The Government of Bangladesh has adopted several strategies and action plans [16, 24] to strengthen the primary healthcare system to address the rising burden of NCDs [25, 26] by introducing community-based and facility-led NCDs management initiatives [27]. To facilitate better NCDs services, the World Health Organization Package of Essential Noncommunicable Disease Interventions for Primary Health Care (WHO-PEN) was adopted [8]. In Bangladesh, the role of the primary healthcare system is key to addressing the rising burden of NCDs because of its nationwide infrastructure, broader service coverage, and cost-effective interventions. Approximately 70% of the population in Bangladesh relies on the primary healthcare system to seek treatment and care [28]. Therefore, precise information about the capacity and limitations of the primary healthcare facilities to deliver NCDs services is essential for effective responses to the NCD epidemic. Recently, a few studies focused on the general and/or disease-specific service availability and readiness of the primary healthcare systems [29–31]. Nonetheless, no studies collectively reported the healthcare facility readiness for all four NCDs (DM, cervical cancer, CRI, and CVD) as prioritized by the WHO. Earlier studies reported combined healthcare facilities' readiness at primary, secondary, and tertiary levels and/or focused on one or two NCDs [29, 30, 32]. However, a holistic analysis of all four WHO-prioritized NCDs at the primary healthcare level in Bangladesh is still a research priority [31, 32]. ‘It is worth mentioning that Bangladesh has a pluralistic health system where a wide range of providers are performing a mixed system of medical practices under the Ministry of Health and Family Welfare from tertiary to primary levels [33]. According to the administrative structures, the country is divided into eight divisions (few districts comprise a division), 64 districts (few sub-districts consist of a district), 490 sub-districts which is locally known as Upazila (several Union Parishad consist of a sub-district), 4553 Union Parishad (several wards consist of a Union Parishad), 40,977 wards (few villages consist of a Ward), and 87,310 villages [34]. The primary healthcare system is established at the sub-district level where a range of public and privately-operated healthcare facilities are functioning. Upazila health complex (UHC) is the first-level public hospital mostly located in the headquarter of the sub-district which provides promotive, preventive, curative, and rehabilitative care. Union-level facilities consisting of 'Union health and family welfare centers (UHFWC)’, 'rural health center (RHD)' and 'Union sub-center' are set up at the Union level (several villages consist of a Union which is the lowest administrative unit) which provide outpatient promotive, and preventive care. Community Clinic (CC) is the lowest level of static healthcare facilities located at the village/wards level which provide outpatients promotive, preventive care. The private facilities (for-profit) at the Upazila level are mostly focused on curative care; while, the NGO facilities (not-for-profit) are focused on basic primary healthcare [33].’. Thus, this study aimed to assess the availability and readiness for WHO-prioritized NCDs in primary healthcare systems in Bangladesh. This study will supplement the information gap, which will help to guide the NCD management efforts in the primary healthcare systems in the country.

## Methodology

### Study design and settings

This was a cross-sectional study of primary healthcare facilities conducted between May and October 2021. Data was collected on the availability of a set of items that are required for NCDs services at the primary healthcare facilities including guidelines and staff, basic equipment, diagnostic facility, and essential medicines. The readiness of the healthcare facilities was assessed for the following NCDs: cervical cancer, CRIs, CVDs, and DM. The specific NCDs were defined according to the diagnosis of the healthcare providers of the respective healthcare facilities. Although the National Cancer Control Strategy and Plan of Action 2009–2015, focused on the prevention and management of commonly prevalent cancer types (e.g., breast, colorectal, esophagus, lung, cervix, lips and oral cavity, etc.), the provision of all these cancers are not available in the primary healthcare level in Bangladesh [35, 36]. Cervical cancer has been gradually increasing over the past years and now is the second most prevalent among women in Bangladesh and subsequently gained greater public health response [37]. The National Cervical Cancer Control Program (2017–22) guideline expanded the provision of cervical cancer services at the primary healthcare level [38]. Considering the scope of services provision, prevalence, health burden, and public health response, this study addressed only cervical cancer readiness [38, 39].

### Study sample and sampling technique

The sample size was calculated using the following formula: (Z^2^*P*d^2^)/(V^2^*P) provided by the Monitoring and Evaluation to ASsess and Use REsults Evaluation (MEASURE) as a sampling manual for the facility surveys [40]. The anticipated proportion of the healthcare facilities, with the attribute of interest P = 50%, design effect d = 1.2, and the relative variance (V^2^) as the square of the relative error taken as 20%, as used by a previous study [41]. This calculation yielded the minimum required sample size of 115 healthcare facilities. Anticipating a 10% non-response rate, we surveyed 126 healthcare facilities. The sample was selected by a multi-stage stratified random sampling technique. Bangladesh is divided into eight administrative divisions [42]. Each division is further divided into several districts, and each district consists of several sub-districts locally known as upazila. Public healthcare facilities are established under administrative units and operate in the following three levels: primary, secondary, and tertiary [43]. The primary care facilities include the Upazila Health Complex (UHC) at the headquarters of a sub-district, union sub-center (USC)/union health center (UHC), family welfare center (FWC) at the union level (hereafter referred to as ‘ULF’ to mean all healthcare facilities at the union level), and community clinic (CC) at the ward level [33]. Along with the public facilities, private and NGO-operated (hereafter referred to as ‘private facilities’) health facilities function within the sub-districts. The total number of healthcare facilities and related information was collected from the Facility Registry database of the Directorate General of Health Services [44]. This study covered 126 healthcare facilities from the following administrative districts of Bangladesh: Cumilla, Jhenaidah, Rajshahi, and Sylhet. Using an electronic structured questionnaire (REDCap), the facility head or management staff member was face-to-face interviewed to collect data.

### Inclusion/exclusion criteria

The health facilities were included based on the following criteria: [1] facilities located at sub-districts level, and (2) facilities providing an NCDs-related service (prevention or management). The facilities were excluded based on the following criteria: (1) did not meet the inclusion criteria; (2) facilities that had less than six months of service; (3) facilities that provided specialized services at the sub-district level (i.e., a tuberculosis clinic); (4) healthcare facilities that had been temporarily established to address the emergency residents (i.e., camp hospitals).

### Data collection team and training

Eight interviewers with a Bachelor of Medicine, Surgery, or Anthropology were involved in the data collection process. Before the interviews, one week of training covering the topic of data collection instruments, filling in the electronic questionnaire, and obtaining information from medical records and data entry into the RedCap software was provided [45]. Additionally, the training focused on the contents of the questionnaire, building rapport, moderating interviews and discussions, taking notes, approaching and inviting interview questions, the organization and function of the primary healthcare system, NCD service delivery package, effective communication with the facility head, and scheduling interviews that enhanced efficiency in the quality data collection. The questionnaire had the following three modules: facility identification, general service availability, and disease-specific readiness. The questionnaire was developed in plain English and then translated into Bengali (the local language). The Bengali version was again translated into English to check the consistency of meaning between versions. The pretest for the questionnaire was conducted, and the necessary feedback was accommodated in the final version. The interview was conducted in Bengali.

### Participants’ consent

Written informed consent was obtained from human participants. Beginning of the interview, the data collectors informed the participants (e.g. the facility head/ management staff) about the purpose of the study. Then the Explanatory Statement was provided by the data collectors to the participants and allowed them to read and ask questions. Upon their agreement to participate, participants were required to read and sign a consent form. The consent form explained the purpose of the study, the freedom to participate, and how participants' information would be used while maintaining their individual/facility information confidential.

### Quality assurance of data collection

To ensure the quality of data collection, the procedure was monitored by the first author throughout the survey. A random consistency check for approximately 5% of the interviewed questionnaires was done by the investigators. Regular group discussions and follow-up meetings were conducted with data collection teams to discuss and share the experience, challenges, and overcoming strategies in conducting interviews. Supportive supervision was provided to the data collectors as required.

### Data collection instrument

Data was collected using a facility survey tool that was developed based on the WHO’s service availability and readiness assessment (SARA) manual (WHO-SARA) [46]. The WHO-SARA tool was designed to generate a set of indicators to determine whether the facilities meet the standardized requirements of general or specific services with reliable quality. This tool offers indicators to monitor and assess several standardized items, including human resources, basic equipment, supplies and technologies, and essential medicine, which are required for general and NCDs-specific service delivery in the healthcare sector. The WHO-SARA is considered a reliable methodology and is widely used to evaluate the readiness of healthcare facilities. The survey questionnaire was designed based on the WHO-SARA methodology, with a slight modification according to the standard set by the Bangladesh Ministry of Health context.

### Outcome measures

The outcome variable in this study is the ‘readiness’ of primary healthcare facilities in terms of four specific NCDs. The readiness variable was rated as an index grouped into four domains as proposed in the WHO SARA methodology: (i) guidelines and staff, (ii) basic equipment, (iii) diagnostic facility, and (iv) essential medicine. Each of these domains has multiple indicators which were measured in nominal scales. In the first domain, there are two indicators: the availability of guidelines, and trained staff for every four NCDs, which was categorized as ‘yes’ for facilities with guidelines, and at least one trained staff for each specific NCDs and ‘no’ otherwise. In both the second and third domains, there are 15 basic equipment and diagnostic items and each of them was categorized as ‘yes’ for the existing facilities, and ‘no’ otherwise. In the fourth domain, 28 essential medicines (a list from Bangladesh health ministry) were categorized as ‘yes’ with facilities reporting the availability of each specific medicine, and ‘no’ otherwise.

### Statistical analysis

Based on the WHO-SARA manual, a descriptive analysis was conducted to define a set of tracer items/readiness indicators for NCDs. The service readiness was assessed into the following four domains: staff and guidelines, equipment and supplies, diagnostic facility, and essential medicine. Based on the SARA tool, the ‘availability’ and ‘readiness’ were categorized as ‘yes’ for facilities with individual items for each specific NCDs and ‘no’ otherwise. In each domain, the scores for the tracer items were calculated and expressed as percentage points (0%–100%). The mean availability service readiness was assessed in the following three levels: (i) determining the tracer score items for four NCDs at each facility level (number of facilities with tracer items*100/the total number of facilities); (ii) calculating the readiness index (RI) of the facility based on the four domains (the mean score of tracer items in each domain); (iii) determining the overall readiness score based on the facility types (the average domain indices for all four domains). The indices were displayed for each of the following facility types: UHCs, ULFs, CCs, and private facilities. These indices were compared to an agreed cut-off threshold of 70%, which means that a facility index below the 70% cut-off was considered not ready to manage NCDs at that level as such previous studies conducted in Bangladesh and elsewhere [29, 47–49]. The summarized scores of the facility-level data are presented as means and standard deviations, and an analysis of variance was performed for determining the significance of the difference between these facilities. Though it was planned in the study protocol [28], multivariable analysis (e.g. multiple binary logistic regression analysis) was not performed to determine the factors related to the overall readiness of the health facilities as the readiness scores were below the cut-off value of 70% for all facilities and domains. This means the outcome measures in this current study failed to meet binary characteristics in order to perform binary logistic regression. All statistical analysis was conducted using SPSS version 22.

### Data storage and management

During the data collection period, the data was saved in the secure REDCap web-based application hosted at Monash University. The application was only accessible to the research team. When the data collection was completed, the data was exported to the IBM SPSS statistical package and saved in the secure faculty-allocated network storage (Monash (S:) drive). Facilities’ identifiers, such as names and addresses, were removed from the main database, saved in a separate secure electronic folder, and not used for the data analysis. Only the research team had access to the electronic databases.

## Results

### Characteristics of healthcare facilities

A total of 126 primary healthcare facilities were assessed, where 77.8% (*n* = 98) were public, and 22.2% (*n *= 28) were private healthcare facilities (Table 1). About 42.1% (*n* = 53) of public healthcare facilities were CCs, 28.6% (*n* = 36) were ULFs, and 7.1% (*n* = 9) were UHCs. Approximately three-fourths of the facilities 75.4% (*n* = 95)) had outpatient clinics, one-fifth had both inpatients and outpatients 21.4% (*n* = 27) services and only 3.2% (*n* = 4) facilities had outpatient services. The average length of services of the facilities was 21.4 (± 13.1) years. Daily six-hour services were available for 76.2% (*n* = 96) of the facilities.

**Table 1:** Characteristics of the health facilities (*n* = 126)

Characteristics	Category	Number of healthcare facilities n (%)
**Management type**		
	Public	98 (77.8)
	Private/Non-government organisations (NGOs)	28 (22.2)
**Facility types**		
	Upazila health complex (UHC)	9 (7.1)
	Private/NGO facilities	28 (22.2)
	Union-level public facilities (ULFs)	36 (28.6)
	Community clinic (CC)	53 (42.1)
**Type of service provided**		
	Outdoor patients (OPT) only	95 (75.4)
	Indoor patients (IPT) only	4 (3.2)
	Both OPT & IPT	27 (21.4)
**Districts**		
	Cumilla	56 (44.4)
	Jhenaidah	17 (13.5)
	Rajshahi	28 (22.2)
	Sylhet	25 (19.8)
**Service in years (operation)**		
	≤ 16 years	49 (38.9)
	17–30 years	51 (40.5)
	> 30 years	26 (20.6)
	Length of service in years, mean and standard deviation	21.4 (13.1)
**Daily service available**		
	≤ 6 h	96 (76.2)
	> 6 h	30 (23.8)

### General service readiness

Table 2 presents the domain-specific general service readiness. The mean tracer item availability score was presented for basic amenities, equipment and supplies, standard precautions, diagnostic capacity, and essential medicine. Among the 126 healthcare facilities, the UHCs had higher availability of general services readiness items than ULFs, CCs, and private facilities. In the context of all levels of primary healthcare facilities, the mean domain scores were as follows: basic amenities (ranged from 62.5% in CCs to 95.2% in UHCs; p = 0.130), equipment and supplies (ranged from 71.4% in CCs to 94.5% in UHCs), standard precautions (ranged from 61.8% in CCs to 94.5% in private facilities; p = 0.009), diagnostic capacity (ranged from 0% in ULFs to 69.4% in UHCs; p < 0.001), and essential medicine (ranged from 9.2% in private facilities to 58.7% in UHCs; p = 0.258).

**Table 2:** General service readiness by health facilities (*n* = 126)

General readiness	Upazila health complex, n (%)	Union-level public facilities, n (%)	Community clinic, n (%)	Private facilities, n (%)	Total n (%)	*P*-value
**Overall**	**9 (7.1)**	**36 (28.6)**	**53 (42.1)**	**28 (22.2)**	**126 (100)**	
**Basic amenities**						
Power	9 (100)	34 (94.4)	49 (92.5)	28 (100)	120 (95.2)	
Improved water source	9 (100)	29 (80.6)	35 (66.0)	28 (100)	101 (80.2)	
Room with privacy	9 (100)	28 (77.8)	28 (52.8)	28 (52.8)	89 (70.6)	
Adequate sanitation facilities	8 (88.9)	30 (83.3)	38 (71.7)	28 (100)	104 (82.5)	
Telephone facilities	9 (100)	28 (77.8)	42 (79.2)	28 (100)	107 (84.9)	
Computer and internet access	9 (100)	24 (66.7)	40 (75.5)	25 (89.3)	98 (77.8)	
Emergency transportation (ambulance)	7 (77.8)	0	0	6 (21.4)	13 (10.3)	
**Mean (± SD)**	95.2 (± 8.7)	68.7 (± 31.4)	62.5 (± 30.1)	80.5 (± 31.2)	71.6 (± 28.1)	0.224
**Basic equipment and supplies**						
Blood pressure apparatus	9 (100)	36 (100)	47 (88.7)	28 (100)	120 (95.2)	
Stethoscope	9 (100)	36 (100)	47 (88.7)	28 (100)	120 (95.2)	
Adult scale	7 (77.8)	19 (52.8)	38 (71.7)	25 (89.3)	89 (70.6)	
Infant scale	8 (88.9)	23 (63.9)	34 (64.2)	24 (85.7)	89 (70.6)	
Thermometer	9 (100)	30 (83.3)	50 (94.3)	28 (100.0)	117 (92.9)	
Light source	9 (100)	13 (36.1)	11 (20.8)	25 (89.3)	58 (46.0)	
**Mean (± SD)**	94.5 (± 9.3)	72.7 (± 26.1)	71.4 (± 27.3)	94.1 (± 6.6)	78.4 (± 19.7)	0.130
**Standard precautions**						
Sterilization equipment	8 (88.9)	30 (83.3)	27 (50.9)	27 (96.4)	92 (73.0)	
Safe disposal of sharps and infectious wastes	9 (100.0)	18 (50.0)	22 (41.5)	24 (85.7)	73 (57.9)	
Waste receptacle	8 (88.9)	27 (75.0)	38 (71.7)	28 (100.0)	101 (80.2)	
Hand‐washing soap and water or alcohol-based hand rub	9 (100.0)	30 (83.3)	44 (83.0)	28 (100.0)	111 (88.1)	
**Mean (± SD)**	94.5 (± 6.4)	72.9 (± 15.8)	61.8(± 19.0)	95.5 (± 6.8)	74.8 (± 12.8)	0.009
**Diagnostic facility**						
Hemoglobin	9 (100.0)	0	14 (26.4)	25 (89.3)	48 (38.1)	
Blood glucose	8 (88.9)	0	33 (62.3)	27 (96.4)	68 (54.0)	
Urine dipstick – protein	4 (44.4)	0	2 (3.8)	23 (82.1)	29 (23.0)	
Urine dipstick – glucose	6 (66.7)	0	2 (3.8)	21 (75.0)	29 (23.0)	
Malaria diagnostic capacity	5 (55.6)	0	0	12 (42.9)	17 (13.5)	
TB microscopy	8 (88.9)	0	0	15 (53.6)	23 (18.3)	
Syphilis RDT	4 (44.4)	0	0	11 (39.3)	15 (11.9)	
Urine pregnancy test	6 (66.7)	18 (50.0)	0	25 (89.3)	49 (38.9)	
**Mean (± SD)**	69.8 (± 22.9)	0 (± 0.0)	13.8 (± 23.4)	68.4 (± 23.0)	26.0 (± 15.1)	< 0.001
**Essential medicines**						
Amitriptyline	0	0	0	0	0	
Amoxicillin	9 (100.0)	35 (97.2)	48 (90.6)	4 (14.3)	96 (76.2)	
Atenolol	7 (77.8)	0	0	0	7 (5.6)	
Captopril	0	0	0	0	0	
Ceftriazone	0	0	0	0	0	
Ciprofloxacin	9 (100.0)	15 (41.7)	0	2 (7.1)	26 (20.6)	
Co‐trimoxazole	7 (77.8)	32 (88.9)	39 (73.6)	3 (10.7)	81 (64.3)	
Diazepam	7 (77.8)	23 (63.9)	0	3 (10.7)	33 (26.2)	
Diclofenac	9 (100.0)	10 (27.8)	0	3 (10.7)	22 (17.5)	
Glibenclamide	0	0	0	0	0	
Omeprazole	8 (88.9)	16 (44.4)	0	7 (25.0)	31 (24.6)	
Paracetamol	9 (100.0)	35 (97.2)	49 (92.5)	7 (25.0)	100 (79.4)	
Simvastatin	0	0	0	0	0	
Salbutamol	9 (100.0)	36 (100.0)	52 (98.1)	7 (25.0)	104 (82.5)	
**Mean (± SD)**	58.7(± 46.2)	40.1 (± 41.9)	25.3 (± 41.9)	9.2 (± 10.0)	28.4 (± 32.6)	0.258

*SD* standard deviation

*P*-values were based on one-way analysis of variance

**Fig. 1:**
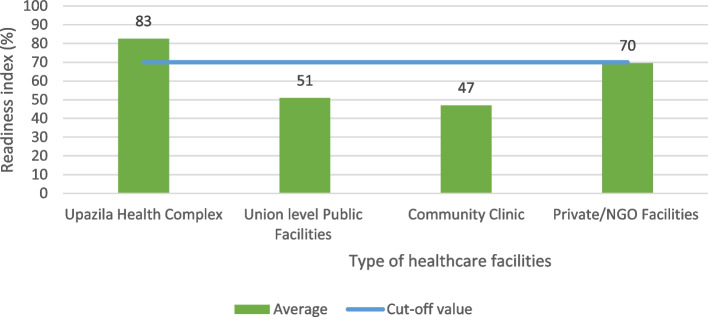
General readiness index scores by healthcare facilities

The overall mean RI for general service availability was highest for the UHCs (83%) and lowest for the CCs (47%) (Fig. 1).

### Readiness index specific to the service for cervical cancer

**Fig. 2:**
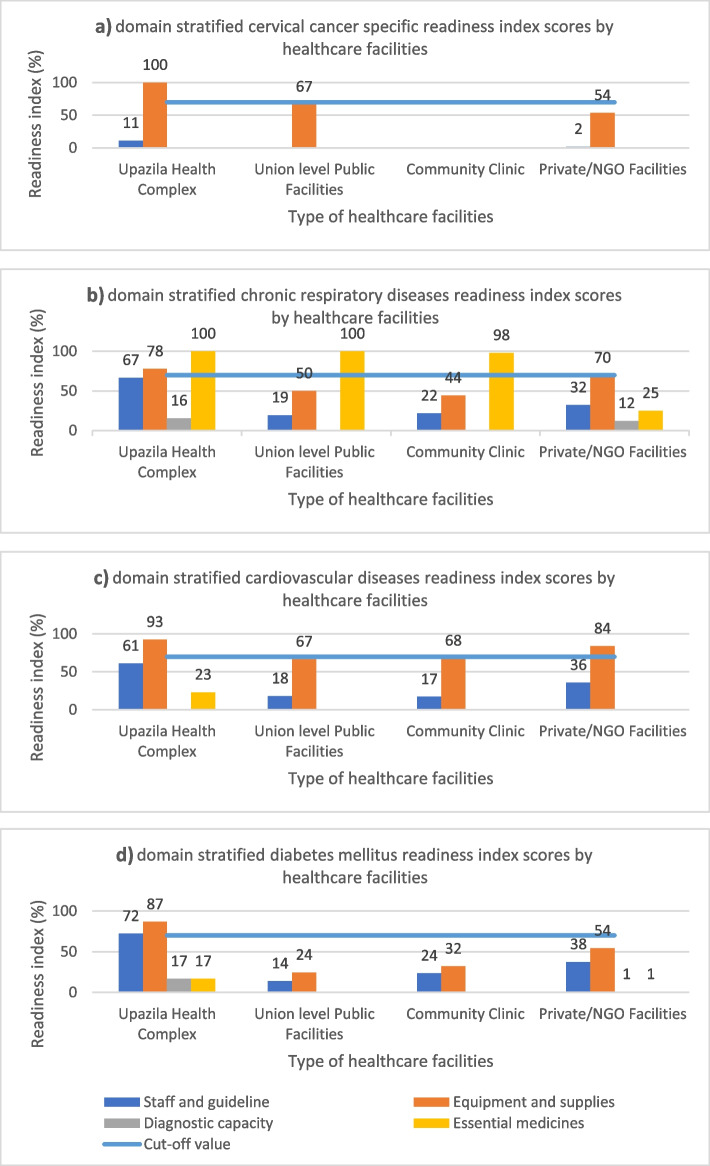
Domain stratified readiness index scores by NCDs (blue line indicates the cut-off value 70%, above which a facility is considered to be ‘ready’ to provide services for NCDs patients)

**Fig. 3:**
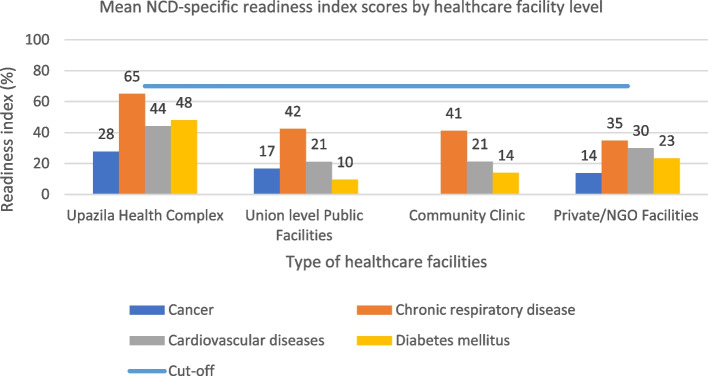
NCDs-specific overall mean readiness index scores by health facility level (blue line indicates the cut-off value of 70%, above which a facility is considered to be ‘ready’ to provide services for NCDs patients)

The RI score of the healthcare facilities for cervical cancer is presented in Table 3. No facilities had guidelines regarding the diagnosis and treatment of cervical cancer. The availability of trained staff was only reported in 11.1% of UHCs. Speculum, the sole tracer item for the basic equipment domain, was available in all UHCs, followed by 66.7% of ULFs, which was followed by 53.6% of private facilities; however, it was unavailable in the CCs. Among the five tracer items of diagnostic facilities, only acetic acid was available at the UHCs and the private facilities (44.4% and 21.4%, respectively), and cytology was available in 10.7% of the private facilities, however, was unavailable at all public facilities. None of the facilities had essential medicine (Fig. 2. a). The overall mean cervical cancer-specific RI score widely varied across the healthcare facilities (ranging from CCs 0% to UHCs 28%) (Fig. 3).

**Table 3:** Cervical cancer-related readiness index scores by facility type (*n* = 126)

Services for cervical cancer	Upazila health complex, n (%)	Union-level public facilities, n (%)	Community clinic, n (%)	Private facilities, n (%)	Totaln (%)	*P*-value
**Overall**	**9 (7.1)**	**36 (28.6)**	**53 (42.1)**	**28 (22.2)**	**126 (100)**	
**Staff and guidelines**						
Guidelines on the diagnosis and treatment of cervical cancer	0	0	0	0	0	
At least one trained staff member (within 24 months)	2 (22.2)	0	0	1 (3.6)	3 (2.4)	
**Mean (± SD)**	11.1 (± 15.7)	N/A	N/A	1.8 (± 2.5)	1.2 (± 1.7)	0.545
**Basic equipment**						
Speculum	9 (100)	24 (66.7)	0	15 (53.6)	48 (38.1)	
**Mean (± SD)**	N/A	N/A	N/A	N/A	N/A	N/A
**Diagnostic facility**						
Acetic Acid	4 (44.4)	0	0	6 (21.4)	10 (7.9)	
Cytology	0	0	0	3 (10.7)	3 (2.4)	
Cancer antigen 15.3 (CA 15.3)	0	0	0	0	0	
Cancer antigen 125 (CA125)	0	0	0	0	0	
Colposcopy	0	0	0	0	0	
**Mean (± SD)**	5.6 (15.7)	N/A	N/A	4.0 (± 8.0)	1.3 (± 2.8)	0.537
**Essential medicines**						
Hydrocortisone	0	0	0	0	0	
Lorazepam	0	0	0	0	0	
Morphin	0	0	0	0	0	
Ondanseron	0	0	0	0	0	
Pheniramine	0	0	0	0	0	
Broad Spectrum Antibiotics	0	0	0	0	0	
**Mean (± SD)**	N/A	N/A	N/A	N/A	N/A	N/A

*SD* standard deviation, *N/A* not applicable

*P*-values were based on one-way analysis of variance

### Readiness index specific to service for chronic respiratory diseases

The score for the tracer indicators for CRI is presented in Table 4. The availability of the guidelines and staff for CRI varied among healthcare facilities (ranging from UHCs at 66.7% to ULFs at 19.4%; p = 0.099). In the context of the basic equipment domain, the highest level of availability was recorded for the blood pressure apparatus (UHCs, ULFs, and private 100%), stethoscope (UHCs, ULFs, and private 100%), and oxygen delivery apparatus (UHCs 88.9%, ULFs, CCs 0%, and private 57.1%). A spacer for inhalers was the least available item for all types of facilities (UHC 22.2%, ULFs and CCs 0%, and 21.4% private). Regarding the diagnostic facility, the highest level of availability was for chest X-rays (UHCs 44.4%, and 46.4% private facilities), which were not available in the ULFs and CCs. No other diagnostic test was available at any facility level. The essential medicine had the highest domain score (UHCs and ULFs 44.7%, CCs 43.9%, private 11.2%; p = 0.965). The highest mean score for CRI was recorded for the domain of basic equipment (84%) in private facilities, and 0% for all diagnostic facilities for all types of healthcare facilities, except UHCs (Fig. 2. b). The overall mean RI score was the highest for UHCs (65%) and lowest for private (35%) (Fig. 3).

**Table 4:** Chronic respiratory illness-related readiness index scores by facility type (*n* = 126)

Services for chronic respiratory illness	Upazila health complex, n (%)	Union-level public facilities, n (%)	Community clinic, n (%)	Private facilities, n (%)	Total n (%)	*P*-value
**Overall**	**9 (7.1)**	**36 (28.6)**	**53 (42.1)**	**28 (22.2)**	**126 (100)**	
**Staff and guidelines**						
Guidelines on the diagnosis and treatment	6 (66.7)	9 (25.0)	10 (18.9)	15 (53.6)	40 (31.7)	
At least one trained staff member (within 24 months)	6 (66.7)	5 (13.9)	13 (24.5)	3 (10.7)	27 (21.4)	
**Mean (± SD)**	66.7 (± 0)	19.5 (± 7.8)	21.7 (± 4.0)	32.2 (± 30.3)	26.6 (± 7.3)	0.099
**Basic equipment**						
Blood pressure apparatus	9 (100)	36 (100)	47 (88.7)	28 (100)	120 (95.2)	
Stethoscope	9 (100)	36 (100)	47 (88.7)	28 (100)	120 (95.2)	
Oxygen delivery apparatus	8 (88.9)	0	0	16 (57.1)	24 (19.0)	
Spacers for inhalers	2 (22.2)	0	0	6 (21.4)	8 (6.3)	
**Mean (± SD)**	77.8 (± 37.4)	50.0 (± 57.7)	44.4 (± 51.2)	69.6 (± 38.0)	53.9 (± 47.9)	0.837
**Diagnostic facility**						
Bacteriology including culture and sensitivity	3 (33.3)	0	0	4 (14.3)	7 (5.6)	
Chest X ray	4 (44.4)	0	0	13 (46.4)	17 (13.5)	
Spirometry test	0	0	0	0	0	
Peak flow meter	0	0	0	0	0	
Sleep test	0	0	0	0	0	
**Mean (± SD)**	15.5 (± 21.6)	N/A	N/A	12.1 (± 20.1)	4.0 (± 6.3)	0.267
**Essential medicines**						
Salbutamol	9 (100.0)	36 (100.0)	52 (98.1)	7 (25.0)	104 (82.5)	
Beclomethasone	0	0	0	0	0	
Prednisolone	0	0	0	0	0	
Hydrocortisone	0	0	0	0	0	
Apinephrine	0	0	0	0	0	
**Mean (± SD)**	20.0 (± 44.7)	20.0 (± 44.7)	19.6 (± 43.9)	5.0 (± 11.2)	16.5 (± 36.9)	0.965

*SD* standard deviation, *N/A* not applicable

*P*-values were based on one-way analysis of variance

### Readiness index specific to service for cardiovascular diseases

The tracer items related to service for CVDs are presented in Table 5. The availability of guidelines and staff for CVDs was highest in UHCs 61.2%, and the lowest in CCs 17.0%). Tracer items under the domain of equipment and supplies, blood pressure apparatus, stethoscopes, and thermometers were mostly available at all types of facilities (ranging from 83.3% to 100%). In comparison, the body mass index (BMI) calculator was the least available item across the facilities (UHCs 88.9% private 28.6%, and unavailable in ULFs and CCs). No diagnostic capacity was available at any level of healthcare facilities. Eight out of thirteen essential medicines were available at the UHCs (ranging from 22.2% to 77.8%), and private facilities (ranging from 1.6% to 5.6%), but unavailable at the ULFs and CCs. The mean RI score of the items was the highest for equipment and supplies (UHCs 93%, ULFs 67%, CCs 68%, private 84%, p = 0.078), and the lowest was zero for the diagnostic capacity at all levels of the primary healthcare facilities (Fig. 2. c). No facility had achieved the cut-off threshold RI score of 70%, which is the minimum requirement to manage CVDs (the breakdown of overall RI score: UHCs 44%, ULFs and CCs 21%, private 30%) (Fig. 3).

**Table 5:** Cardiovascular diseases-related readiness index scores by facility type (*n* = 126)

Services for cardiovascular diseases	Upazila health complex, n (%)	Union-level public facilities, n (%)	Community clinic, n (%)	Private facilities, n (%)	Total n (%)	*P*-value
**Overall**	**9 (7.1)**	**36 (28.6)**	**53 (42.1)**	**28 (22.2)**	**126 (100)**	
**Staff and guidelines**						
Guidelines on the diagnosis and treatment	6 (66.7)	9 (25.0)	13 (24.5)	15 (53.6)	43 (34.1)	
At least one trained staff member (within 24 months)	5 (55.6)	4 (11.1)	5 (9.4)	5 (17.9)	19 (15.1)	
**Mean (± SD)**	61.2 (± 7.8)	18.1 (± 9.8)	17.0 (± 10.7)	35.8 (± 25.2)	24.6 (± 13.4)	0.124
**Basic equipment**						
Blood pressure apparatus	9 (100)	36 (100)	47 (88.7)	28 (100)	120 (95.2)	
Stethoscope	9 (100)	36 (100)	47 (88.7)	28 (100)	120 (95.2)	
Adult scale	7 (77.8)	19 (52.8)	38 (71.7)	25 (89.3)	89 (70.6)	
Thermometer	9 (100)	30 (83.3)	50 (94.3)	28 (100.0)	117 (92.9)	
BMI calculator	8 (88.9)	0	0	8 (28.6)	16 (12.7)	
**Mean(± SD)**	92.6 (± 9.1)	66.7 (± 37.8)	67.9 (± 35.2)	83.9 (± 27.8)	72.9 (± 31.7)	0.708
**Diagnostic facility**						
Blood lipids	0	0	0	0	0	
Electrolytes potassium	0	0	0	0	0	
**Mean (± SD)**	N/A	N/A	N/A	N/A	N/A	N/A
**Essential medicines**						
Atenolol	7 (77.8)	0	0	0	7 (5.6)	
Digoxin	4 (44.4)	0	0	0	4 (3.2)	
Enalapril	3 (33.3)	0	0	0	3 (2.4)	
Furosemide	4 (44.4)	0	0	0	4 (3.2)	
Glyceryl trinitrate	0	0	0	0	0	
Isosorbide dinitrate	0	0	0	0	0	
Nifedipine	2 (22.2)	0	0	0	2 (1.6)	
Methyldopa	3 (33.3)	0	0	0	3 (2.4)	
Procainamide	0	0	0	0	0	
Propranolol	2 (22.2)	0	0	0	2 (1.6)	
Spironolactone	2 (22.2)	0	0	0	2 (1.6)	
Verapamil	0	0	0	0	0	
Warfarin	0	0	0	0	0	
**Mean (± SD)**	23.1 (± 23.8)	N/A	N/A	N/A	1.7 (± 1.7)	< 0 .001

*SD* standard deviation, *N/A* not applicable

*P*-values were based on one-way analysis of variance

### Readiness index specific to service for DM

The availability of tracer items for the service that is specific to DM is presented in Table 6. The guidelines for diagnosis and treatment were available across all facility types (UHCs 66.7%, ULF 16.7%, CCs 28.3%, and private 53.6%). The availability of the required basic equipment, such as blood pressure apparatus, stethoscope, and height board/stadiometer were in more than half of the facilities. Regarding the tracer items in the domain of diagnostic capacity, the blood glucose test was available in the UHCs (88.9%), CCs (62%), and private facilities (96.4%). However, there was no capacity for ULFs. Metformin was the only available essential medicine at the UHCs (66.7%) (Fig. 2. d). No facilities had an overall mean score above the cut-off threshold of 70% for the diabetes service (UHCs 48%, ULF 10%, CCs 14%, and private 23%) (Fig. 3).

**Table 6:** Diabetes mellitus-related readiness index scores by facility type (*n* = 126)

Services for diabetes mellitus	Upazila health complex, n (%)	Union-level public facilities, n (%)	Community clinic, n (%)	Private facilities, n (%)	Total n (%)	*P*-value
**Overall**	**9 (7.1)**	**36 (28.6)**	**53 (42.1)**	**28 (22.2)**	**126 (100)**	
**Staff and guidelines**						
Guidelines on the diagnosis and treatment	6 (66.7)	6 (16.7)	15 (28.3)	15 (53.6)	42 (33.3)	
At least one trained staff member (within 24 months)	7 (77.8)	4 (11.1)	10 (18.9)	6 (21.4)	27 (21.4)	
**Mean (± SD)**	72.3 (± 7.8)	13.9 (± 4.0)	23.6 (± 6.6)	37.5 (± 22.8)	27.4 (± 8.4)	0.027
**Basic equipment**						
Blood pressure apparatus	9 (100)	36 (100)	47 (88.7)	28 (100)	120 (95.2)	
Stethoscope	9 (100)	36 (100)	47 (88.7)	28 (100)	120 (95.2)	
Weighing scale	7 (77.8)	19 (52.8)	38 (71.7)	25 (89.3)	89 (70.6)	
Height board/stadiometer	8 (88.9)	25 (69.4)	47 (88.7)	27 (96.4)	107 (84.9)	
BMI calculator	8 (88.9)	0	0	8 (28.6)	16 (12.7)	
**Mean (± SD)**	86.7 (± 5.0)	24.4 (± 34.0)	32.1 (± 44.3)	54.3 (± 35.3)	38.7 (± 36.0)	0.684
**Diagnostic facility**						
Blood glucose (HbA1c/OGTT)	8 (88.9)	0	33 (62.3)	27 (96.4)	68 (54.0)	
Urine dipstick – protein	4 (44.4)	0	2 (3.8)	23 (82.1)	29 (23.0)	
Urine dipstick – glucose	6 (66.7)	0	2 (3.8)	21 (75.0)	29 (23.0)	
**Mean (± SD)**	66.7 (± 22.3)	N/A	23.2 (± 33.6)	84.5 (± 10.9)	33.3 (± 17.9)	0.003
**Essential medicines**						
Glibenclamide	0	0	0	0	0	
Gliclazide	0	0	0	0	0	
Insulin	0	0	0	0	0	
Metformin	6 (66.7)	0	0	1 (3.6)	7 (5.6)	
**Mean (± SD)**	16.7 (± 33.4)	N/A	N/A	0.9 (± 1.8)	1.4 (± 2.8)	0.473

*SD* standard deviation, *N/A* not applicable

*P*-values were based on one-way analysis of variance

## Discussion

This study assessed the readiness of the primary healthcare facilities to address the four NCDs in Bangladesh. To the best of our knowledge, this is the first study to assess the readiness of both public and private primary healthcare for the four NCDs recommended by the WHO. Therefore, the findings of this study are comprehensive and it extensively reports the status of service availability and gaps regarding each of the NCDs. The readiness for the general services was above the 70% cut-off threshold value for the domains of basic amenities, equipment and supplies, and standard precautions for all types of healthcare facilities. However, only UHCs meet this threshold for items related to diagnostic facilities and essential medicine. All levels of primary healthcare facilities failed to meet the globally accepted threshold value to manage NCDs. Among the NCDs, disease-specific readiness for CRI and DM were higher compared to cervical cancer and CVDs at the primary healthcare facilities. The service-specific items in the domains of the staff and guidelines, essential medicine, and diagnostic facility were highly insufficient for cervical cancer and CVDs in the ULFs and CCs.

The shortage of human resources and respective treatment guidelines was commonly reported in and across the facilities. The availability of at least one trained staff was highest for DM (77.8%) at the UHCs, whereas it was the lowest for cervical cancer (3.6%), and there was no trained staff at UHFs and CCs. A recent systematic review noted that the preparedness of DM is relatively higher than cancer at the primary healthcare level [50]. A previous study in Bangladesh reported relatively higher domain scores for the guidelines and trained staff for cervical cancer [29]. The possible reason for this could be this study included secondary and tertiary hospitals such as district hospitals, maternity hospitals, and specialized hospitals that are usually better equipped. The shortage of human resources at healthcare facilities is a common feature of the health system in low- and middle-income counties [51, 52]. Healthcare systems, including India [53, 54], Thailand [55], and Uganda [56] are struggling to respond to the increasing demands for NCD services at the primary care level [57]. Similarly, Bangladesh is facing a shortage of trained healthcare staff, which was notably high for CVDs- and cervical cancer-specific services [29, 58]. The lack of trained front-line staff at the primary level facilities negatively affects the NCDs outcomes in many countries [50, 59–62]. Although there is little scope for deploying disease-specific trained healthcare staff in the primary care facilities [32], routine NCDs-related training for front-line staff (e.g., the Community Health Care Providers, Sub Assistant Community Medical Officers, Health Assistants) and nurses would be effectively useful in managing NCDs.

The equipment and diagnostic capacity of the healthcare facilities are the preconditions of comprehensive and quality NCDs services. The availability of equipment and diagnostic capacity items was noticeably low at all facility levels in Bangladesh which is concordant with a recent review [63]. The review showed the readiness score for basic equipment for NCDs was relatively low (ranged 29.2% to 51.2% based on WHO-PEN standards) facilities in low-and-middle-income countries which is concordant with our findings; indicating a large proportion of facilities lack basic equipment and diagnostic capacity required to deliver the services [63]. Previous studies reported the highest availability of blood pressure apparatus and stethoscopes (> 90%) in Bangladesh [31, 32] and Tanzania [64, 65], which is supported by this current study. However, the diagnostic capacity related to cervical cancer and CVD is noticeably low, which indicates a lower readiness level. Our study showed a slightly higher availability of bacteriology (including culture and sensitivity) and chest X-rays, whereas sleep tests and spirometry were unavailable at all primary healthcare levels [30].

Essential medicines are the critical requirement for effective NCDs services and care. Individual, and review studies conducted in similar settings highlighted the shortage of essential medicine impeding the management of NCDs [12, 29, 58, 63]. Although the essential medicine list for four NCDs included 78 types of medicine, Bangladesh’s national guidelines included 30 different medicines from this list [66]. Nevertheless, the availability of the listed essential medicine for all four NCDs was noticeably low. The availability of medicine was the lowest for cervical cancer and highest for CRI. Noticeably, no essential medicine for cervical cancer, CVD, or DM was available at the ULFs, CCs, and private facilities. The shortage and inconsistent supply of essential medicines categorically lowered the readiness scores. More importantly, studies reported that the lack of essential medicine at the primary healthcare facilities resulted in private purchases with high out-of-pocket healthcare expenditures [12]. Unlike infectious diseases/acute conditions, NCDs treatment requires long-term medication support and has a higher out-of-pocket cost, which leads to medical nonadherence or household impoverishment [67, 68]. Although this current study’s aims did not allow us to determine the extent of medical nonadherence resulting from the shortage of essential medicine, we hypothesize that it would have significantly affected people with a lower socioeconomic status.

### Strengths and limitations

The strength of this study is that it holistically investigated the readiness of all four WHO-prioritized NCDs at all levels of primary healthcare facilities. Furthermore, a random selection of 126 primary healthcare facilities across Bangladesh, which includes both public and private facilities is another strength of this study. However, there are a few limitations that should be acknowledged. One limitation is that the readiness indicators were assessed according to the WHO-SARA methodology, which solely focused on the supply-side aspects, e.g., infrastructure, supplies and commodities, and human resources. This methodology would not have fully identified the dynamic interaction and specific factors that influence the broader health system components [28]. In addition, some of the collected information was the respondents' verbal responses, which were not possible to verify. Therefore, it might have resulted in bias. In the context of Bangladesh, it was not possible to validate the respondents’ (facility heads/designated personnel) responses separately because of restricted access to the healthcare facilities’ information records.

## Conclusion

Our findings suggest that primary healthcare facilities' readiness for NCDs remains remarkably low. Shortage of staff and guidelines, diagnostic capacity, and essential medicine are significant challenges for managing cervical cancer and CVDs at all facility levels. The capacity of primary healthcare facilities needs to be significantly improved to manage the NCDs, with a focus on increasing trained personnel and essential medical supplies and improving diagnostic facilities.

## Data Availability

The data used and analyzed during this research are not publicly available due to ethical restrictions, and data confidentiality. Data are available upon reasonable request from researchers who meet the criteria for access to confidential data. Interested parties may contact the first author (md.kabir@monash.edu) for further inquiries in this regard.

## Abbreviations

CC: Cervical cancer
CCs: Community clinics
CRIs: Chronic respiratory diseases
CVDs: Cardiovascular diseases
DM: Diabetes mellitus
NCDs: Non-communicable diseases
RI: Readiness index
UHCs: Upazila health complexes
ULFs: Union-level facilities
USC: Union sub-center
WHO-PEN: World Health Organization Package of Essential Noncommunicable Disease Interventions for Primary Health Care
WHO-SARA: World Health Organization Service Availability and Readiness Assessment

## References

[1] World Health Organization. Noncommunicable diseases: Key facts 2021 [Available from: https://www.who.int/news-room/fact-sheets/detail/noncommunicable-diseases.

[2] Wang Y, Wang J (2020). Modelling and prediction of global non-communicable diseases. BMC Public Health.

[3] NCD Alliance. The Financial Burden of NCDs 2022 [Available from: https://ncdalliance.org/why-ncds/the-financial-burden-of-ncds.

[4] World Health Organization. Global action plan for the prevention and control of noncommunicable diseases 2013–2020. 2013.

[5] Beaglehole R, Epping-Jordan J, Patel V, Chopra M, Ebrahim S, Kidd M (2008). Improving the prevention and management of chronic disease in low-income and middle-income countries: a priority for primary health care. Lancet.

[6] Bennett JE, Stevens GA, Mathers CD, Bonita R, Rehm J, Kruk ME (2018). NCD Countdown 2030: worldwide trends in non-communicable disease mortality and progress towards Sustainable Development Goal target 3.4. The Lancet.

[7] World Health Organization. Package of essential noncommunicable (PEN) disease interventions for primary health care in low-resource settings. 2010.

[8] Zaman M, Ullah A, Bhuiyan M, Karim M (2016). Noncommunicable disease prevention and control situation in a primary health care setting of Bangladesh: design and baseline findings of an intervention. Chronic Dis Int.

[9] Laatikainen T, Inglin L, Collins D, Ciobanu A, Curocichin G, Salaru V (2020). Implementing package of essential non-communicable disease interventions in the Republic of Moldova-a feasibility study. Eur J Public Health.

[10] Krishnan A, Mathur P, Kulothungan V, Salve HR, Leburu S, Amarchand R (2021). Preparedness of primary and secondary health facilities in India to address major noncommunicable diseases: results of a National Noncommunicable Disease Monitoring Survey (NNMS). BMC Health Serv Res.

[11] Abolhassani N, Santos-Eggimann B, Chiolero A, Santschi V, Henchoz Y (2019). Readiness to accept health information and communication technologies: a population-based survey of community-dwelling older adults. Int J Med Inform.

[12] Elias MA, Pati MK, Aivalli P, Srinath B, Munegowda C, Shroff ZC (2017). Preparedness for delivering non-communicable disease services in primary care: access to medicines for diabetes and hypertension in a district in south India. BMJ Glob Health.

[13] Katende D, Mutungi G, Baisley K, Biraro S, Ikoona E, Peck R (2015). Readiness of Ugandan health services for the management of outpatients with chronic diseases. Trop Med Int Health.

[14] Mutale W, Bosomprah S, Shankalala P, Mweemba O, Chilengi R, Kapambwe S (2018). Assessing capacity and readiness to manage NCDs in primary care setting: gaps and opportunities based on adapted WHO PEN tool in Zambia. PLoS ONE [Electronic Resource].

[15] Van Minh H, Do YK, Bautista MA, Tuan Anh T (2014). Describing the primary care system capacity for the prevention and management of non-communicable diseases in rural Vietnam. Int J Health Plann Manage.

[16] El-Saharty S, Ahsan KZ, Koehlmoos TL, Engelgau MM. Tackling noncommunicable diseases in Bangladesh: now is the time: World Bank Publications; 2013.

[17] Alam D, Robinson H, Kanungo A, Hossain MD, Hassan M. Health Systems Preparedness for responding to the growing burden of non-communicable disease-a case study of Bangladesh. Health Policy & Health Finance knowledge Hub The Nossal Institute for Global Health The University of Melbourne. 2013:1–25.

[18] Chowdhury AM, Bhuiya A, Chowdhury ME, Rasheed S, Hussain Z, Chen LC (2013). The Bangladesh paradox: exceptional health achievement despite economic poverty. Lancet.

[19] World Health Organization. Bangladesh: World Health Organization; [cited 2020 August 13]. Available from: https://www.who.int/workforcealliance/countries/bgd/en/.

[20] Zaman M, Ullah A, Bhuiyan M (2016). Noncommunicable disease prevention and control situation in a primary health care setting of Bangladesh: design and baseline findings of an intervention. Chronic Dis Int.

[21] Khalequzzaman M, Chiang C, Choudhury SR, Yatsuya H, Al-Mamun MA, Al-Shoaibi AAA (2017). Prevalence of non-communicable disease risk factors among poor shantytown residents in Dhaka, Bangladesh: a community-based cross-sectional survey. BMJ Open.

[22] Osman FA (2008). Health policy, programmes and system in Bangladesh. South Asian survey.

[23] Islam A, Biswas T (2014). Health system in Bangladesh: challenges and opportunities. Am J Health Res.

[24] Hussain A. Reducing Dietary Related Risks associated with Non-Communicable Diseases in Bangladesh (RDRNCD): Successes and Gaps in Reducing NCDs: Policy Recommendations.

[25] Biswas T, Pervin S, Tanim MIA, Niessen L, Islam A (2017). Bangladesh policy on prevention and control of non-communicable diseases: a policy analysis. BMC Public Health.

[26] Rawal LB, Kanda K, Biswas T, Tanim MI, Poudel P, Renzaho AMN (2019). Non-communicable disease (NCD) corners in public sector health facilities in Bangladesh: a qualitative study assessing challenges and opportunities for improving NCD services at the primary healthcare level. BMJ Open.

[27] Rawal L, Jubayer S, Choudhury SR, Islam SMS, Abdullah AS (2020). Community health workers for non-communicable diseases prevention and control in Bangladesh: a qualitative study. Glob Health Res Policy.

[28] Kabir A, Karim MN, Billah B (2021). Primary healthcare system readiness to prevent and manage non-communicable diseases in Bangladesh: a mixed-method study protocol. BMJ Open.

[29] Rakhshanda S, Dalal K, Chowdhury HA, Mayaboti CA, Paromita P, Rahman AKMF (2021). Assessing service availability and readiness to manage cervical cancer in Bangladesh. BMC Cancer.

[30] Paromita P, Chowdhury HA, Mayaboti CA, Rakhshanda S, Rahman A, Karim MR (2021). Assessing service availability and readiness to manage Chronic Respiratory Diseases (CRDs) in Bangladesh. PLoS ONE.

[31] Islam MR, Laskar SP, Macer D (2016). A Study on service availability and readiness assessment of non-communicable diseases using the WHO tool for Gazipur District in Bangladesh. Bangladesh J Bioeth.

[32] Biswas T, Haider MM, Das Gupta R, Uddin J (2018). Assessing the readiness of health facilities for diabetes and cardiovascular services in Bangladesh: a cross-sectional survey. BMJ Open.

[33] Ministry of Health & Family Welfare of Bangladesh. Health Bulletin 2019 Dhaka, Bangladeshhttps://dghs.gov.bd/images/docs/Publicaations/Health%20Bulletin%202019%20Print%20Version%20(2)-Final.pdf: Diretorate of Health Services; 2019 [cited 2020 November 12]. Available from: https://dghs.gov.bd/images/docs/Publicaations/Health%20Bulletin%202019%20Print%20Version%20(2)-Final.pdf.

[34] Bangladesh Bureau of Statistics. Population and Housing Census 2011: Socio-economic and Demographic Report Dhaka, Bangladesh. 2014.

[35] Ministry of Health & Family Welfare of Bangladesh. National Cancer Control Strategy and Plan of Action 2009–15. Dhaka, Bangladesh.

[36] Hussain SA, Sullivan R (2013). Cancer control in Bangladesh. Jpn J Clin Oncol.

[37] Hoque MR, Haque E, Karim MR (2021). Cervical cancer in low-income countries: a Bangladeshi perspective. Int J Gynecol Obstetr.

[38] Ministry of Health and Family Welfare. National Strategy for Cervical Cancer Prevention and Control in Bangladesh (2017–2022). Dhaka, Bangladesh; 2017

[39] Nessa A, Chowdhury SB, Fatima P, Kamal M, Sharif M, Azad AK (2020). Cervical cancer screening program in Bangladesh. Bangladesh J Obstetr Gynaecol.

[40] Turner AG, Angeles G, Tsui AO, Wilkinson M, Magnani R. Sampling manual for facility surveys for population, maternal health, child health and STD programs in developing countries. 2000.

[41] Shawon MSR, Adhikary G, Ali MW, Shamsuzzaman M, Ahmed S, Alam N (2018). General service and child immunization-specific readiness assessment of healthcare facilities in two selected divisions in Bangladesh. BMC Health Serv Res.

[42] Administrative geography of Bangladesh [Available from: https://en.wikipedia.org/wiki/Administrative_geography_of_Bangladesh.

[43] World Health Organization. Bangladesh health system review: Manila: WHO Regional Office for the Western Pacific; 2015.

[44] Ministry of Health and Family Welfare. Facility Registry: Government of People's Republic of Bangladesh 2021 [Available from: http://facilityregistry.dghs.gov.bd/.

[45] Harris PA, Taylor R, Thielke R, Payne J, Gonzalez N, Conde JG (2009). Research electronic data capture (REDCap)—a metadata-driven methodology and workflow process for providing translational research informatics support. J Biomed Informa.

[46] World Health Organization. Service availability and readiness assessment (SARA): an annual monitoring system for service delivery: reference manual. World Health Organization; 2013.

[47] Albelbeisi AH, Albelbeisi A, El Bilbeisi AH, Takian A, Akbari-Sari A (2020). Capacity of Palestinian primary health care system to prevent and control of non-communicable diseases in Gaza Strip, Palestine: A capacity assessment analysis based on adapted WHO-PEN tool. Int J Health Plann Manage.

[48] Chowdhury HA, Paromita P, Mayaboti CA, Rakhshanda S, Rahman FN, Abedin M (2022). Assessing service availability and readiness of healthcare facilities to manage diabetes mellitus in Bangladesh: Findings from a nationwide survey. PLoS ONE.

[49] Mutale W, Bosomprah S, Shankalala P, Mweemba O, Chilengi R, Kapambwe S (2018). Assessing capacity and readiness to manage NCDs in primary care setting: gaps and opportunities based on adapted WHO PEN tool in Zambia. PLoS ONE.

[50] Kabir A, Karim MN, Islam RM, Romero L, Billah B (2022). Health system readiness for non-communicable diseases at the primary care level: a systematic review. BMJ Open.

[51] Liu JX, Goryakin Y, Maeda A, Bruckner T, Scheffler R (2017). Global health workforce labor market projections for 2030. Human Res Health.

[52] Kabir A, Karim N, Billah B (2022). Preference and willingness to receive non-communicable disease services from primary healthcare facilities in Bangladesh: a qualitative study. BMC Health Serv Res.

[53] Pakhare A, Kumar S, Goyal S, Joshi R (2015). Assessment of primary care facilities for cardiovascular disease preparedness in Madhya Pradesh. India BMC Health Serv Res.

[54] Panda R, Mahapatra S, Persai D (2018). Health system preparedness in noncommunicable diseases: Findings from two states Odisha and Kerala in India. J Family Med Prim Care.

[55] Aekplakorn W, Suriyawongpaisal P, Sirirassamee B (2005). Assessment of capacity for cardiovascular disease control and prevention in Thailand: a qualitative study. Southeast Asian J Trop Med Public Health.

[56] Musinguzi G, Bastiaens H, Wanyenze RK, Mukose A, Van Geertruyden JP, Nuwaha F (2015). Capacity of health facilities to manage hypertension in Mukono and Buikwe Districts in Uganda: challenges and recommendations. PLoS ONE [Electronic Resource].

[57] Kabir A, Karim MN, Billah B (2022). Health system challenges and opportunities in organizing non-communicable diseases services delivery at primary healthcare level in Bangladesh: a qualitative study. Front Public Health.

[58] Biswas T, Haider MM, Das Gupta R, Uddin J (2018). Assessing the readiness of health facilities for diabetes and cardiovascular services in Bangladesh: a cross-sectional survey. BMJ Open.

[59] Katende D, Mutungi G, Baisley K, Biraro S, Ikoona E, Peck R (2015). Readiness of Ugandan health services for the management of outpatients with chronic diseases. Trop Med Int Health.

[60] Duong DB, Minh HV, Ngo LH, Ellner AL (2019). Readiness, Availability and utilization of rural vietnamese health facilities for community based primary care of non-communicable diseases: a crosssectional survey of 3 provinces in Northern Vietnam. Int J Health Policy Manag.

[61] Kien VD, Van Minh H, Giang KB, Ng N, Nguyen V, Tuan LT (2018). Views by health professionals on the responsiveness of commune health stations regarding non-communicable diseases in urban Hanoi, Vietnam: a qualitative study. BMC Health Serv Res.

[62] Meiqari L, Nguyen T-P-L, Essink D, Wright P, Scheele F (2020). Strengthening human and physical infrastructure of primary healthcare settings to deliver hypertension care in Vietnam: a mixed-methods comparison of two provinces. Health Policy Plann.

[63] Albelbeisi AH, Albelbeisi A, El Bilbeisi AH, Taleb M, Takian A, Akbari-Sari A (2021). Public sector capacity to prevent and control of noncommunicable diseases in twelve low- and middle-income countries based on WHO-PEN standards: a systematic review. Health Serv Insights.

[64] Adinan J, Manongi R, Temu GA, Kapologwe N, Marandu A, Wajanga B (2019). Preparedness of health facilities in managing hypertension & diabetes mellitus in Kilimanjaro, Tanzania: a cross sectional study. BMC Health Serv Res.

[65] Bintabara D, Mpondo BCT (2018). Preparedness of lower-level health facilities and the associated factors for the outpatient primary care of hypertension: Evidence from Tanzanian national survey. PLoS ONE.

[66] Islam SMS, Islam MT, Islam A, Rodgers A, Chow CK, Naheed A (2017). National drug policy reform for noncommunicable diseases in low-resource countries: an example from Bangladesh. Bull World Health Organ.

[67] Kabir A, Datta R, Raza SH, Maitrot MRL (2019). Health shocks, care-seeking behaviour and coping strategies of extreme poor households in Bangladesh's Chittagong Hill tracts. BMC Public Health.

[68] Kabir A, Maitrot MRL, Kiyu A (2018). Exploring the effects of health shocks on anti-poverty interventions: Experience of poor beneficiary households in Bangladesh. Cogent Med.

